# Reviews

**Published:** 1879-08

**Authors:** 


### REVIEWS.

---

**Lectures on Practical Surgery.** By H. H. LOLAND, M. D., Professor of Practical and Clinical Surgery in the University of California. Second Edition. Illustrated. Philadelphia: Lindsay & Blakiston. 1879.

This work we infer is designed as a class text book in the medical department of the University of California, and is well adapted to this object. As a report of lectures it is quite complete, forming a text book of quite extensive scope. The conclusions of the author are quite unlike our own on some subjects; for instance, he says: "Resections of the hip-joint present no difficulty in the execution. I have performed the operation myself three times, but have not been satisfied with the result, and I do not think that I could under ordinary circumstances be induced to repeat it. In children the limb may be saved; but it is attached to the pelvis by the muscles, and is nothing better than a dangling mass of bones and flesh. Recently I have resected the femur, the details and results I will give elsewhere. Amputation of the hip is much better than resection. When the head of the thigh-bone is exposed, there frequently and indeed almost universally, exists caries of the acetabulum, which it becomes necessary to remove. This of course destroys the articulating surfaces, and consequently the use of the lower extremity."

This is quite unlike our own experience, and we quote it so as to show both sides. Other conclusions of the author strike us as remarkable, but space prevents extended mention. J. F. M.

---

**Notes on the Treatment of Skin Diseases.** By ROBERT LIVEING, A. M., F. R. C. S. William Wood & Co., 27 Great Jones Street, New York. 1878.

The work consists of short notes on the etiology and treatment of skin diseases, and were prepared for the students in cutaneous medicine, at the Middlesex Hospital, but the little volume is adapted to all students of skin diseases, and will be appreciated by general practitioners as well as by students of medicine. J. F. M.

---

**Lectures on Bright's Disease of the Kidneys.** By J. M. CHARCOT, and Translated by HENRY B. MILLARD, M. D., A. M. New York: William Wood & Co. 1878.

This work is comprised in seven lectures, giving the normal anatomy of the kidney with physiological considerations, and a summary of views of Bright's disease, nephritis, and the lesions produced in all its various forms, the scarlatinous kidney, amyloid kidney, &c., &c. The work is written with great care and precision, embodies the whole subject and affords us a clear, comprehensive, condensed, practical treatise upon the important subject of Bright's disease. No work could afford us more instruction, or outline the practical features of the disease more clearly. More voluminous treatises are published, but we believe none more complete or practical. J. F. M.

---

**An Atlas of Human Anatomy**; illustrating most of the ordinary dissections and many not usually practiced by the student, accompanied by an explanatory text. By RICKMAN JOHN GODLEY, M. S., F. R. C. S. Philadelphia: Lindsay & Blakiston.

These plates are very beautiful colored representations of the the normal tissues as seen after dissection, and are invaluable for both the anatomist and surgeon, representing the relation and appearance of parts with astonishing accuracy. The work is in parts and sold at the low price of $2.50 each part. J. F. M.

---

**Diseases of the Throat and Nasal Cavities.** By Dr. CARL SEILER. Philadelphia: Henry C. Lea. Buffalo: T. H. Butler.

It is an admirable little book, containing 150 pages and 35 illustrations. We recommend it most heartily to the busy practitioner, who has no time or inclination to study the larger special works. It presents in small compas the methods and instruments used in laryngoscopy and rhinoscopy, and the diagnosis and treatment of the most common affections of the upper air passages. M.

